# First‐trimester plasma extracellular heat shock proteins levels and risk of preeclampsia

**DOI:** 10.1111/jcmm.17674

**Published:** 2023-03-31

**Authors:** Claudia Melina Robellada‐Zárate, Janelly Estefania Luna‐Palacios, Carlos Agustín Zapata Caballero, Juan Pablo Acuña‐González, Irlando Lara‐Pereyra, Diego Iván González‐Azpeitia, Ricardo Josué Acuña‐González, Elsa Romelia Moreno‐Verduzco, Héctor Flores‐Herrera, Mauricio Osorio‐Caballero

**Affiliations:** ^1^ Departamento de Ginecología y Obstetricia Instituto Nacional de Perinatología “Isidro Espinosa de los Reyes” Ciudad de México Mexico; ^2^ Departamento de Inmunobioquimica Instituto Nacional de Perinatología “Isidro Espinosa de los Reyes” Ciudad de México Mexico; ^3^ Departamento de Matemáticas, Facultad de Ciencias Universidad Nacional Autónoma de México Ciudad de México Mexico; ^4^ Departamento de Ginecología, Hospital General de Zona 252 Instituto Mexicano del Seguro Social Atlacomulco Mexico; ^5^ Departamento de Ginecología Hospital General de León León Mexico; ^6^ Subdirección de Servicios Auxiliares de Diagnóstico Instituto Nacional de Perinatología “Isidro Espinosa de los Reyes” Ciudad de México Mexico; ^7^ Departamento de Salud Sexual y Reproductiva Instituto Nacional de Perinatología “Isidro Espinosa de los Reyes” Ciudad de México Mexico

**Keywords:** extracellular heat shock proteins, preeclampsia, pregnancy

## Abstract

Preeclampsia (PE) occurs annually in 8% of pregnancies. Patients without risk factors represent 10% of these. There are currently no first‐trimester biochemical markers that accurately predict PE. An increase in serum 60‐ and 70‐KDa extracellular heat shock proteins (*e*Hsp) has been shown in patients who developed PE at 34 weeks. We sought to determine whether there is a relationship between first‐trimester *e*Hsp and the development of PE. This was a prospective cohort study performed at a third level hospital in Mexico City from 2019 to 2020. *e*Hsp levels were measured during the first‐trimester ultrasound in singleton pregnancies with no comorbidities. First‐trimester *e*Hsp levels and biochemical parameters of organ dysfunction were compared between patients who developed preeclampsia and those who did not. All statistical analyses and model of correlation (*r*) between *e*Hsp and clinical parameter were performed using bootstrapping R‐software. *p*‐values <0.05 were considered significant. The final analysis included 41 patients. PE occurred in 11 cases. *e*Hsp‐60 and *e*Hsp−70 were significantly higher at 12 weeks in patients who developed PE (*p* = 0.001), while *e*Hsp‐27 was significantly lower (*p* = 0.004). Significant differences in first‐trimester *e*Hsp concentration suggest that these are possible early biomarkers useful for the prediction of PE.

## 1 INTRODUCTION

Preeclampsia (PE) is a heterogeneous, multisystemic hypertensive disorder of pregnancy of variable severity that is associated with 60,000 deaths per year worldwide.^1^ Preeclampsia complicates from 2 to 8% of pregnancies and continues to be one of the leading causes of maternal^2^, ^3^ and foetal death.^4^ It is associated with complications such as foetal growth restriction (FGR)^5^, ^6^, ^7^ and placental abruption.^5^, ^8^, ^9^

Preeclampsia development is divided in two stages.^10^, ^11^ During the first stage, inefficient cytotrophoblast invasion early in the first trimester causes inadequate and incomplete remodelling of the spiral arteries, with consequent high vessel resistance and low blood flow which cause placental ischemia.^12^, ^13^ The measurement of blood flow through uterine arteries reflect these changes and, coupled with maternal blood pressure, are the only validated first‐trimester prediction models.^14^

During the second stage of this abnormal placentation, there is a proangiogenic and antiangiogenic imbalance,^15^, ^16^ including soluble Fms‐like tyrosine kinase‐1 (sFlt‐1), vascular endothelial growth factor (VEGF) and placental growth factor (PIGF).^17^, ^18^, ^19^, ^20^ The decrease in these proteins in plasma is correlated with the severity of PE and with the development of adverse pregnancy outcomes^21^, ^22^, ^23^ attributed to an increase in pro‐inflammatory cytokines.^24^, ^25^ These serum markers and their indices (sFlt‐1/PIGF ratio) are only used in prediction models later on in pregnancy.^26^

Extracellular heat shock proteins (*e*Hsp), first described by Ferruccio Ritossa in 1962, are molecules stable at high temperature present in both prokaryotic and eukaryotic cells.^27^ These molecules are expressed during cellular stress and regulate cellular homeostasis,^28^ proliferation and differentiation of the professional immune system cells.^29^, ^30^, ^31^

Extracellular heat shock proteins have been classified as being of high (60, 70, 90, 100‐kDa) or low (20, 27‐kDa) molecular weight.^32^, ^33^ When released into the extracellular space, *e*Hsp function as cell‐to‐cell mediators.^27^, ^34^ *e*Hsp‐60 (HSPD1; heat shock protein family D member 1A) and eHsp‐70 (HSPA1A; heat shock protein family A member 1A) stimulate pro‐inflammatory cytokines production,^27^, ^34^ while *e*Hsp‐27 (HSPB1; heat shock protein family B [small] 1) has an important anti‐inflammatory function.^35^, ^36^, ^37^ Their concentration has also been shown to increase in the serum of patients with severe trauma,^37^, ^38^ inflammatory processes^39^, ^40^ and PE.^41^, ^42^, ^43^ Therefore, *e*Hsp have been used as sensible indicators of the physiological status during the onset and resolution of different pathological conditions.^44^, ^45^

Recently, our research group has confirmed significantly higher levels of *e*Hsp‐60 and *e*Hsp‐70 in patients with PE compared with same gestational age controls, as well as a linear relationship between their concentration and levels of inflammatory cytokines (IL‐1β and TNFα), uric acid, creatinine, lactate dehydrogenase and liver transaminases (AST and ALT)^41^; *e*Hsp concentrations in the first trimester of pregnancy were not assessed. The aim of this study was to determine whether first‐trimester serum levels of *e*Hsp are associated with the development of PE and if there is a correlation between *e*Hsp and serum biomarkers of end‐organ dysfunction present in PE.

## 2 MATERIAL AND METHODS

### 2.1 Ethics statements

This study was reviewed and approved by the National Institute of Perinatology Ethics and Research Committees (registration number 212250–3210091). All patients were explained the purpose of the study, and informed consent was obtained.

### 2.2 Study design and patients

We performed a prospective cohort study. The data were derived from screening in women attending their first‐trimester routine ultrasound at the National Institute of Perinatology in Mexico City, México, between February 2019 and January 2020.

### 2.3 Clinical definitions and inclusion criteria

Inclusion criteria were singleton pregnancies without a history of PE who were able to attend a first‐trimester ultrasound and have their blood drawn for *e*Hsp measurement. Patients were excluded from the study when the amount of blood collected for the quantification of *e*Hsp was insufficient (plasma <1200 μl), as well as patients diagnosed with infectious/inflammatory morbidities, including sepsis, intraamniotic infection/inflammation, premature rupture of membranes and placental abruption or prior PE in other pregnancies. These patients received follow‐up until delivery, and careful care was taken to identify patients who developed PE. The diagnosis of PE was based on the criteria of the American College of Obstetricians and Gynecologists (ACOG), which define it as the presence of new‐onset hypertension (≥140 mmHg systolic or ≥90 mmHg diastolic) after 20‐week gestation with coexistence of either significant proteinuria (spot urine protein/creatinine >0.3 mg/mg or >300 mg/day or at least 2+ on dipstick testing) or signs of maternal organ dysfunction (platelet count <100 × 10^9^/L), serum creatinine concentrations >1.1 mg/dl or a doubling of the serum creatinine concentration in the absence of other renal disease, elevated blood concentrations of liver transaminases to twice normal concentration, pulmonary oedema or new‐onset headache unresponsive to medication and not accounted for by alternative diagnoses or visual symptoms.^46^

### 2.4 Obtaining blood samples and biochemical assays

During the first ultrasound visit, which is held at 11–13 weeks of gestation, peripheral blood samples (5 ml) were collected in K_2_‐EDTA vacutainer tubes (Becton‐Dickinson; NJ, USA) and centrifuged at 294 *g* for 5 min. Plasma was recovered in Eppendorf tubes and stored at −80°C. Commercial ELISA kits were used to quantify the levels of *e*Hsp‐27 (DYC‐1580; R&D Systems, Minneapolis, MN, USA), *e*Hsp‐60 (DYC1800‐2; R&D Systems) and *e*Hsp‐70 (DYC1663‐2; R&D System) according to the commercial manufacturer's specifications as previously reported by our research group.^41^ Standard curves were calculated from 31.3 to 2000 ng/ml, 1.25 to 80 ng/ml and 312.5 to 20,000 pg/ml, respectively. The following sensitivity values for each protein were calculated: 50, 0.70 and 150.0 respectively. Uric acid, creatinine, lactate dehydrogenase (LDH), aspartate aminotransferase (AST) and alanine aminotransferase (ALT) were measured in a VITROS 4600 Chemistry System (Ortho Clinical Diagnostics, Ratiran, NJ) using specific kits according to the commercial manufacturer's guidelines and has been previously reported by our research group.^41^

### 2.5 Data analysis

All assays were independently replicated at least three times. Laboratory parameters, clinical data and *e*Hsp concentrations between healthy and PE patients were compared using Student's *t*‐test. The Spearman rank correlation (*r*) between *e*Hsp and clinical parameters was compared by bootstrapping analysis for small samples.^47^ The data are presented as means with SD. Statistical analysis was carried out using GraphPad Prism version 8.0 (GraphPad Software, San Diego, CA; USA). A *p*‐value <0.05 was considered significant. All statistical analyses were performed using the R software statistical computing version 4.1.2 (R Foundation, Vienna, Austria; https://www.R‐project.org/).

## 3 RESULTS

A total of 48 women were followed up after *e*Hsp measurement during the first‐trimester ultrasound. Of these, seven were eventually excluded for the following reasons: premature rupture of membranes (*n* = 4), placental abruption (*n* = 2) and foetal growth restriction (FGR) without PE (*n* = 1). In total, 41 women with singleton pregnancies were included in this study, of which 11 developed PE. Of these, three developed early PE and eight late PE. One patient part of late PE group developed HELLP syndrome and two patients FGR + PE.

### 3.1 Demographic data of the study population

Table 1 shows the demographic and clinical characteristics of these patients compared with those who did not develop PE. No significant differences were found with respect to age (31.3 ± 7.6 vs. 30.4 ± 8.5; *p* = 0.753), weight (67.7 ± 15.6 kg vs. 64.06 ± 14.5 kg; *p* = 0.501), height (1.5 ± 0.05 vs. 1.56 ± 0.04) and body mass index (27.4 ± 6.06 kg/m^2^ vs. 26.2 ± 5.4 kg/m^2^ *p* = 0.541). Systolic and diastolic blood pressure increased 1.3‐fold in patients who developed preeclampsia (*p* = 0.001). Gestational age at delivery (37.8 ± 3.1 weeks vs. 35.9 ± 4.2 weeks; *p* = 0.31) and birthweight decreased (3039 ± 499 kg vs. 2442 ± 803.8 kg; *p* = 0.032) significantly with respect to the healthy group (Table 1).

**TABLE 1:** Comparison of maternal characteristics at the time of admission, at the development of preeclampsia and neonatal at the delivery between the two groups of pregnant patients

Parameters	Healthy (*n* = 30)	PE (*n* = 11)	CI	*p‐value*
At the time of admission				
Age (years)	31.3 ± 7.6	30.4 ± 8.5	0.0683–0.0649	0.753
Weight (Kg)	67.6 ± 15.6	64.06 ± 14.5	0.0229–0.0265	0.501
Height (m)	1.5 ± 0.05	1.56 ± 0.04	0.0264–0.0298	0.933
BMI at sampling (Kg/m^2^)	27.4 ± 6.06	26.2 ± 5.4	0.0358–0.0323	0.541
Blood pressure at time of PE				
Systolic (mmHg)	109.9 ± 8.7	146.7 ± 18.2	0.0902–0.0866	0.001
Diastolic (mmHg)	68.9 ± 6.9	90.5 ± 12.9	0.0921–0.0885	0.001
At delivery				
Gestational age (weeks)	37.8 ± 3.1	35.9 ± 4.2	0.3148–0.3184	0.31
Birthweight (Kg)	3039 ± 499.0	2442 ± 803.8	0.0894–0.0929	0.032

*Note*: Data are shown as the mean ± SD and were analysed using the bootstrapping with 10,000‐times repeated. Statistically significant difference in <0.05 was considered.

Abbreviations: BMI, body mass index; CI, confidence intervals.

### 3.2 Biochemical parameters

Table 2 shows the values of serum biochemical parameters at the time of PE diagnosis. No significant differences were found in haemoglobin (*p* = 0.986), haematocrit (*p* = 0.781), leukocytes (*p* = 0.258), creatinine (*p* = 0.195), LDH (*p* = 0.307), GOT (*p* = 0.269), GPT (*p* = 0.063), alkaline phosphatase (*p* = 0.105), glucose (*p* = 0.623), urinary creatinine (*p* = 0.665), 24 h urine protein (*p* = 0.315) and urinary protein/creatinine (*p* = 0.352); however, the values of uric acid (*p* = 0.012), platelets (*p* = 0.047) and urinary total protein (*p* = 0.008) increased and the concentration of bilirubin decreased significantly in patients with PE with compared with the group of healthy patients (Table 2).

**TABLE 2:** Clinical and biochemical parameters at the time of PE diagnosis

Parameter	Healthy (*n* = 30)	PE (*n* = 11)	CI	*p‐value*
Uric acid (mg/dl)	4.23 ± 1.15	5.42 ± 1.2	0.0395–0.0360	0.012
Haemoglobin (g/dl)	13.0 ± 1.37	13.2 ± 1.38	0.0484–0.0449	0.986
Haematocrit (%)	39.55 ± 3.9	39.3 ± 4.49	0.0487–0.0452	0.781
Leukocytes (10^3^/mm^3^)	7.4 ± 1.5	8.7 ± 1.4	0.0119–0.0153	0.258
Platelets (10^3^/mm^3^)	228.2 ± 61.7	299.2 ± 119.2	0.1353–0.1319	0.047
Creatinine (mg/dl)	0.49 ± 0.12	0.56 ± 0.17	0.1057–0.1022	0.195
LDH (UI/L)	206.8 ± 106.8	170.1 ± 54.1	0.1862–0.1827	0.307
GOT (U/L)	24.4 ± 7.5	31.7 ± 21.0	0.2568–0.2533	0.269
GPT (IU/L)	21.16 ± 10.5	37.6 ± 24.34	0.1792–0.1755	0.063
Alkaline phosphatase (IU/L)	132.4 ± 49.6	163.8 ± 49.3	0.0575–0.0539	0.105
Glucose (mg/ml)	78.5 ± 9.7	79.8 ± 6.4	0.0176–0.0210	0.623
Urinary creatinine (mg/ml)	61.4 ± 26.76	55.36 ± 34.1	0.0630–0.0594	0.665
Urinary total protein (mg/ml)	13.62 ± 4.2	63.3 ± 74.15	0.3652–0.3617	0.008
24 h urine protein (mg/dl)	269.5 ± 151.5	392.6 ± 161.8	0.1108–0.1072	0.315
Urinary protein/creatinine ratio	0.2968 ± 0.21	0.59 ± 0.39	0.2232–0.2197	0.352
Total bilirubin (mg/dl)	0.54 ± 0.39	0.28 ± 0.14	0.2781–0.2746	0.012

*Note*: Data are shown as the mean ± SD and were analysed using the bootstrapping with 10,000‐times repeated. Statistically significant difference in <0.05 was considered.

Abbreviation: CI, confidence intervals.

### 3.3 Extracellular heat shock protein quantification in serum

**FIGURE 1:**
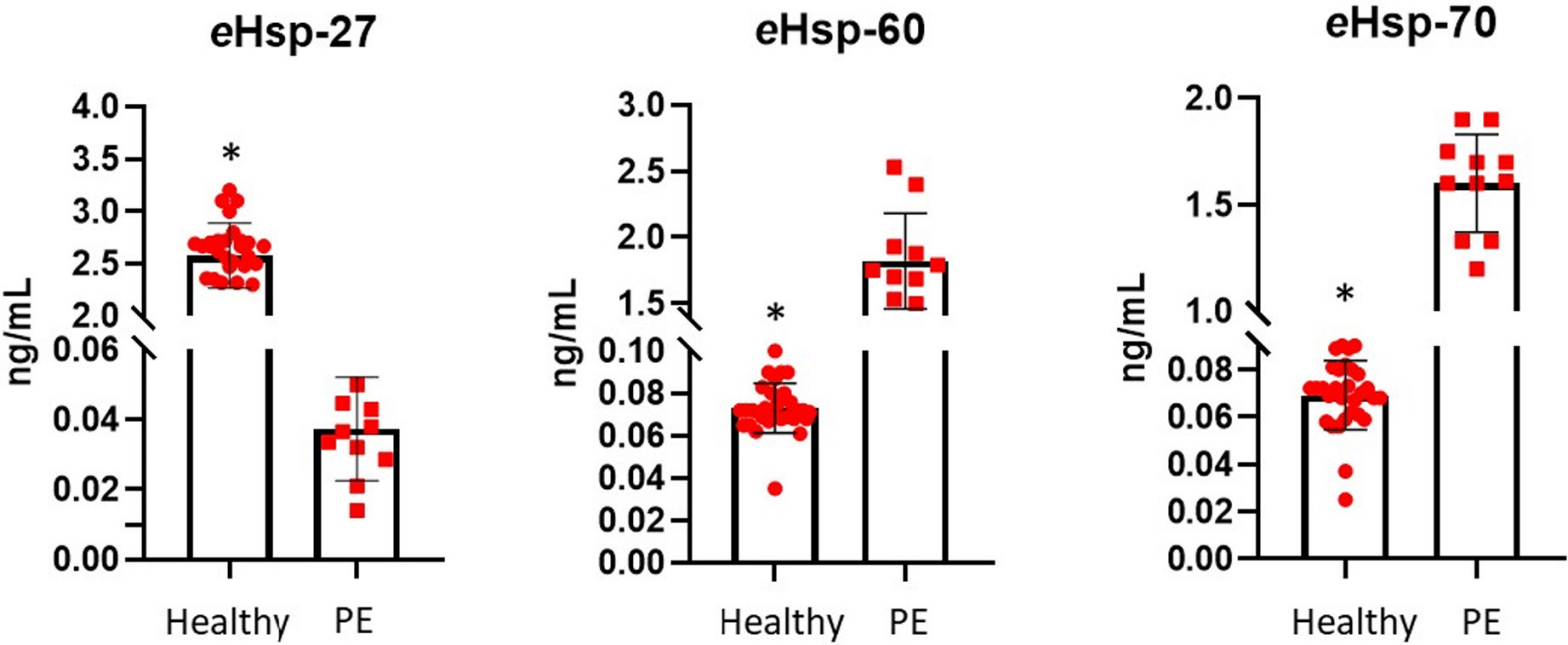
Comparison in the secretion of *e*Hsp‐27, *e*Hsp‐60 and *e*Hsp‐70. Healthy patients at term (*n* = 30) and patients with PE (*n* = 11). Concentration is expressed as ng/ml. Data represent the mean ± SD. Statistical difference was observed between both groups *p ≤ 0.0001.

Figure 1 shows concentrations of plasma *e*Hsp‐27, *e*Hsp‐60 and *e*Hsp‐70 at 12 weeks of gestation in both populations. The concentration of *e*Hsp‐27 was 69.4‐fold lower (2.57 ± 0.310 vs 0.037 ± 0.014 pg/ml; *p* = 0.0001) in patients who developed PE, while *e*Hsp‐60 and *e*Hsp‐70 were 24.9‐fold (0.073 ± 0.011 vs 1.82 ± 0.36 pg/ml; *p* = 0.0001) and 23.2‐fold higher (0.069 ± 0.014 vs 1.60 ± 0.22 pg/ml; *p* = 0.0001).

Furthermore, our results showed a significant correlation between *e*Hsp‐60 levels and UA (*r* = 0.6468; *p* = 0.0336), LDH (*r* = 0.8245; *p* = 0.0036), GOT (*r* = 0.7677; *p* = 0.0143), GPT (*r* = 0.7804; *p* = 0.0071), creatinine (*r* = 0.8888; *p* = 0.0007) and platelets (*r* = 0.6104; *p* = 0.0632) in PE patients. Finally, a significant correlation coefficient was also found between *e*Hsp‐70 levels and UA (*r* = 0.6295; *p* = 0.0487), LDH (*r* = 0.7247; *p* = 0.0205), GOT (*r* = 0.6584; *p* = 0.0210), GPT (*r* = 0.6772; *p* = 0.0239), creatinine (*r* = 0.6436; *p* = 0.0323) and platelets (*r* = 0.6052; *p* = 0.0431) in PE patients (Table 3).

**TABLE 3:** Correlations between *e*Hsp and biochemical parameters in PE patients.

*e*Hsp	Biochemical	AUC (CI)	Coefficient of correlation	
			*r*	*p‐value*
−60	UA	1.0 (0.0337–0.0313)	0.6468	0.0336
	LDH	0.962 (0.0042–0.0036)	0.8245	0.0036
	GOT	0.566 (0.0147–0.0132)	0.7677	0.0143
	GPT	0.758 (0.0072–0.0065)	0.7804	0.0071
	Creatinine	0.758 (0.0007–0.0006	0.8888	0.0007
	Platelets	0.758 (0.0673–0.0631)	0.6104	0.0632
−70	UA	0.772 (0.0492–0.0446)	0.6295	0.04871
	LDH	0.747 (0.0215–0.0194)	0.7247	0.0205
	GOT	0.967 (0.0222–0.0208)	0.6584	0.0210
	GPT	0.920 (0.0245–0.0227)	0.6772	0.0239
	Creatinine	0.911 (0.0324–0.0303)	0.6436	0.0323
	Platelets	0.758 (0.0438–0.0408)	0.6052	0.0431

*Note*: Data are shown as the mean ± SD and were analysed using the bootstrapping with 10,000‐times repeated. Statistically significant difference in <0.05 was considered.

Abbreviations: AUC, area under curve; CI, confidence intervals.

## 4 DISCUSSION

Inefficient cytotrophoblast invasion and abnormal placentation promotes an imbalance between angiogenic and antiangiogenic factors.^48^, ^49^ Deficiencies in this cellular communication lead to the development of PE.^50^, ^51^ It has been accurately shown that *e*Hsp are an important part of the innate immune response and regulation of physiological processes,^10^, ^31^ in response to infections of human foetal membranes,^50^ prelabour rupture of membranes and preterm birth.^52^, ^53^, ^54^ Recently, our research group has shown that *e*Hsp‐60 and *e*Hsp‐70 are increased in patients with PE at 34 weeks of gestation compared with healthy patients.^41^ In the same study, there was a significant correlation between both *e*Hsp‐60 and *e*Hsp‐70 and biochemical markers of organ dysfunction. Nevertheless, the association between these markers and the development of PE has not been studied in the first trimester.

In this study, we noted that pregnant women who subsequently developed PE had a downregulation of *e*Hsp‐27 and upregulation of *e*Hs‐60 and *e*Hsp‐70 at 12‐week‐gestation compared with women who did not develop PE. We also found a high correlation of *e*Hsp‐60 and *e*Hsp‐70 with respect to PE biochemical markers of organ dysfunction.

Recently, Martin et al.^55^ showed that the *e*Hsp‐27 concentration increases up to twofold in the first and second trimesters in patients with PE compared with healthy term patients. Also, this was associated with a decrease in placental protein‐13, which is involved in normal placentation.^56^, ^57^ Unfortunately, in this study, the functional role of *e*Hsp‐27 in the regulation of the inflammatory response was not addressed.

Álvarez‐Cabrera et al. have shown a relationship between *e*Hsp‐60 and *e*Hsp‐70 with the increase in IL‐Iβ and TNFα in patients with PE compared with healthy patients at term.^41^ During preeclampsia development, maternal inflammatory cytokines (IL‐Iβ, IL‐6, IL‐8 and TNFα) are activated,^58^, ^59^, ^60^ reducing the expression of *e*Hsp‐27 and increasing the *e*Hsp‐60 and *e*Hsp‐70 levels.^58^

**FIGURE 2:**
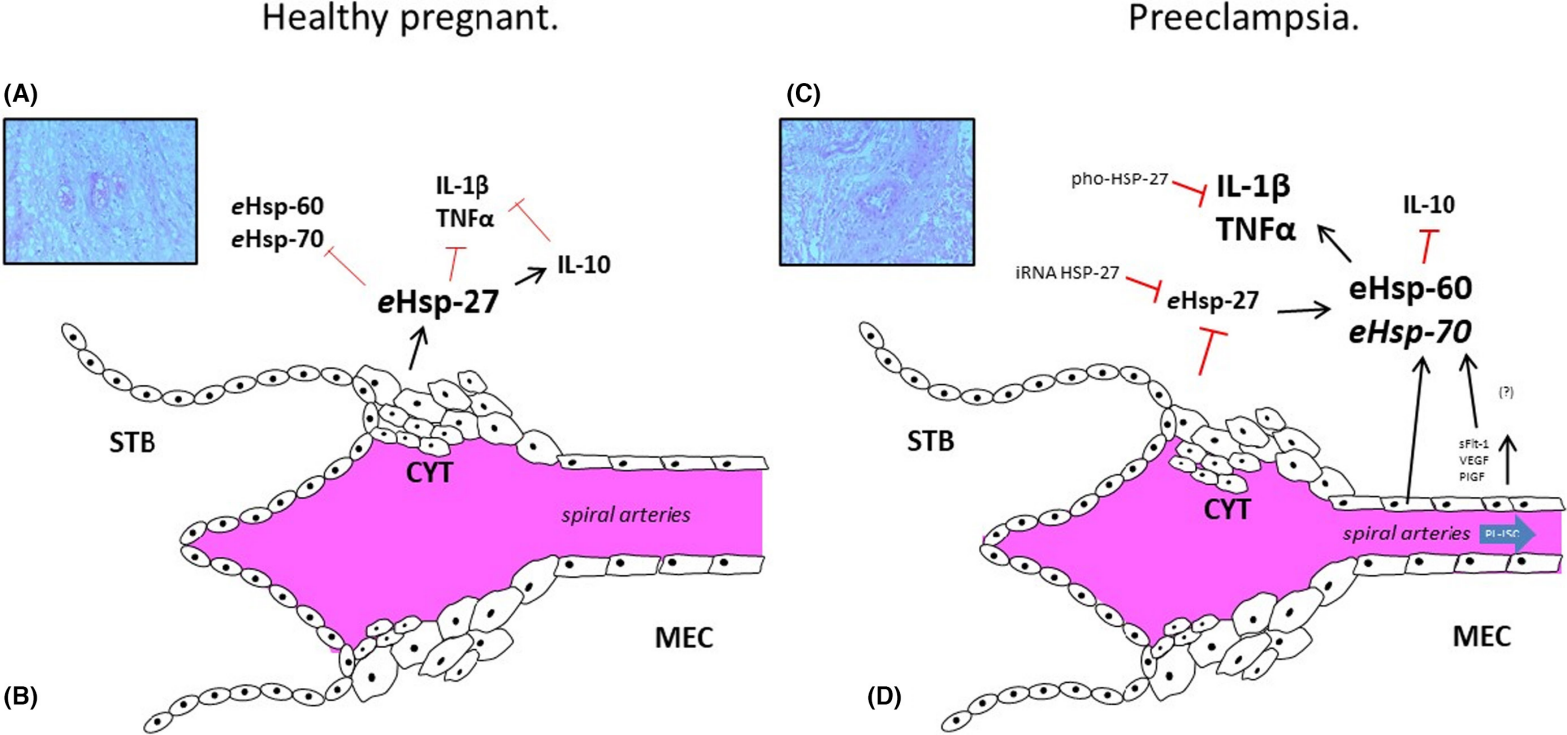
Interaction model of *e*Hsp and inflammatory cytokines in normal pregnancy and preeclampsia. (A) Tissue of human health, decidual cells are observed normal and arterioles without alterations. At 12‐weeks' gestation the cytotrophoblast secreted *e*Hsp‐27^55^ and active production of anti‐inflammatory interleukin (IL)‐10 via intracellular p38MAPK pathways which reduce the secretion of proteins associated with oxidative stress, apoptosis, and the inflammatory response.^68^ (B) However, this secretion profile changes in the development of preeclampsia. (C) Tissue of human preeclampsia decidual vasculopathy with fibrinoid necrosis are shown. (D) Inadequate remodelling by the cytotrophoblast invasion of spiral arteries reducing blood flow causing placental ischemia (PLA‐ISC)^12^, ^13^ promoting the secretion of sFlt‐1,^16^ VEGF^20^ and PIGF.^18^ This environment promotes deregulation between *e*Hsp‐60, *e*Hsp‐70 and *e*Hsp‐27 (Figure 1). The importance of Hsp has been shown by cells transfected with phosphorylated form Hsp‐27 (pho‐Hsp‐27) reduced the IL‐1β and TNFα signalling pathway,^61^ and by the small interfering RNA degrades Hsp‐27 mRNA increasing IL‐1 β concentration.^62^

Figure 2 shows a interaction model between *e*Hsp and inflammatory cytokines in both healthy pregnant patients and in the development of preeclampsia. Li et al.^61^ have shown that in human mononuclear cells (Thp1) transfected with the phosphorylated form of Hsp‐27 reduces the concentration of TNFα, IL‐1β and IL‐6 induced by LPS (500 ng/ml) in a dose‐dependent manner and that this effect is mediated by the inhibition of the MyD88 and IRF3 signalling pathway.^61^

Using peripheral blood mononuclear cells (PBMC), Hadadi et al.^62^ have shown that by inhibiting the expression of Hsp‐27 with small interfering RNA the secretion of IL‐1β increases up to twofold compared with the control group. Our results show that in patients at 12 weeks of gestation without a history of preeclampsia, the levels of *e*Hsp‐27 decrease, and there is an upregulation of *e*Hsp‐60 and *e*Hsp‐70 in patients who will develop PE compared with healthy ones at term (Figure 1).

Extensive research has led to the identifying several potential biomarkers for the detection of preeclampsia^63^, ^64^, ^65^, ^66^; however, their sensitivity and specificity for early detection are low.^67^ It has recently been shown that development of PE is associated with increased levels of *e*Hsp‐70, *e*Hsp‐90 and TNFα in blood and plasma.^41^ Our findings provide new evidence and support previous results showing that *e*Hsp‐60 and *e*Hsp‐70 are increased in the development of PE.

In the next phase of the study, we intend to follow‐up with the patients and take blood samples from the first, second and third trimesters of pregnancy. At the time of delivery, we will take a sample of the placental tissue and assess how it relates with eHsp levels at systemic level. Three months after the baby is born, a final blood sample will be drawn from patients. These results will allow us to establish: (1) *e*Hsp levels throughout pregnancy in healthy patients; (2) changes in *e*Hsp levels, and how they related with the severity of PE (mild, moderate, severe and HELLP); (3) if 3 months after delivery *e*Hsp levels return to baseline values in the PE group, as expected; and (4) how systemic *e*Hsp levels related to placental tissue. Finally, we will make the association between Hsp with markers sFlt‐1, VEGF and PIGF involved with PE.

Some limitations in the present study must be addressed. First, the small sample size may limit statistical power. Second, early‐onset and late‐onset preeclampsia were grouped together, as well as PE with and without severe features. Further studies analysing *e*Hsp levels within these subclassifications may reach stronger conclusions. Third, as there is no cut‐off point for normal or abnormal *e*Hsp levels, a potential relationship was not explored. Finally, as all participants in our study were Mexican, caution should be exercised when extrapolating our findings to other populations.

## 5 CONCLUSION

Our findings suggest that the inflammatory response in patients who develop PE affects *e*Hsp levels as early as 12‐week gestation. Therefore, a case can be made in favour of developing a novel screening strategy including the measurement of *e*Hsp‐60 and *e*Hsp‐70 during the first trimester as a reliable biomarker of early development of PE. As mentioned previously, the high sensitivity of these proteins as biomarkers provide a potential link with organ malfunction.

In this study, we have confirmed that there are significantly higher levels of *e*Hsp‐60 and *e*Hsp‐70 in patients with PE compared with same gestational age controls. Nevertheless, we did not measure *e*Hsp levels at the time of PE diagnosis, so further studies are needed to assess *e*Hsp changes over time.

## AUTHOR CONTRIBUTIONS

**Claudia Melina Robellada‐Zárate:** Data curation (equal); formal analysis (equal); methodology (equal); writing – review and editing (equal). **Janelly Estefania Luna‐Palacios:** Methodology (equal). **Carlos Agustín Zapata‐Caballero:** Data curation (equal); formal analysis (equal); writing – review and editing (equal). **Juan Pablo Acuña‐González:** Formal analysis (equal). **Irlando Lara‐Pereyra:** Formal analysis (equal). **Diego Ivan González‐Azpeitia:** Formal analysis (equal). **Ricardo Josué Acuña‐González:** Formal analysis (equal). **Elsa Romelia Moreno‐Verduzco:** Formal analysis (equal); methodology (equal). **Héctor Flores‐Herrera:** Conceptualization (equal); formal analysis (equal); funding acquisition (equal); writing – original draft (equal). **Mauricio Osorio‐Caballero:** Conceptualization (equal); data curation (equal); formal analysis (equal); supervision (equal); writing – review and editing (equal).

## FUNDING INFORMATION

This study was supported by the Instituto Nacional de Perinatología ‘Isidro Espinosa de los Reyes’, Ciudad de México, México (grant number 212250–3210091 to HF‐H). The institute was not involved in any stage of the study and has no conflict of interest with the content of the manuscript. The authors CMR‐Z, CAZ‐C, IL‐P, DIG‐A, RJA‐G, HF‐H and MO‐C paid for the publication of the article.

## CONFLICT OF INTEREST

The authors have no conflicts of interests with the research or the authorship publication. All authors carefully read the final version of the manuscript and gave their consent for journal submission.

## Data Availability

All of the relevant information from the study is described in the manuscript.
